# Predicting 90-day falls and unplanned medical visits in nursing home residents using rapid nursing scores including calf circumference, grip strength, Timed Up and go (TUG) test, weight change, and appetite: a prospective observational cohort study protocol

**DOI:** 10.3389/fpubh.2026.1812292

**Published:** 2026-08-06

**Authors:** Dan Wang, Xianhu Yang, Jiaxin Yang, Hongna Gao, Shubai Chen, Huijing Sun, Fei Liu, Guoqiang Ma, Yalin Mao, Daoxia Sun, Haina Cui

**Affiliations:** 1 Binzhou Polytechnic University, Binzhou, China; 2 Shandong Jingren Elderly Care Service Co., Ltd, Binzhou, China; 3 Handan First Hospital, Handan, China; 4 Hebei Medical University, Hebei, China; 5 The Philippine Women’s University, Manila, Philippines; 6 Binzhou People’s Hospital, Jianxiang, Binzhou, China

**Keywords:** Nursing homes, older adults, falls, unplanned medical visits, muscle function, nutritional status, risk assessment

## Abstract

**Background:**

Falls are the main health and safety threat faced by older adults in nursing homes, which can cause serious injuries and increase the risk of death. At present, there is a lack of simple fall risk screening tools that are applicable to the care practices of nursing homes and take into account both muscle function and nutritional status. Develop and validate a rapid scoring tool based on care accessibility indicators [calf circumference, grip strength, up/Walk timing Test (TUG), weight change and appetite] to predict the composite risk of falls or unplanned medical visits (emergency visits or unplanned hospitalizations) among older adults in care institutions within 90 days.

**Methods:**

This study was a prospective observational cohort study. It is planned to recruit approximately 500 older adults aged 65 and above from 10 nursing homes in Binzhou City, Shandong Province from September 2026 to November 2026 by using the convenience sampling method. Sociodemographic, clinical characteristics (history of falls, comorbidities), cognitive function and activities of daily living data were collected, and calf circumference, grip strength, TUG test, weight change (compared with at admission) and appetite (using a simplified nutritional appetite questionnaire) were measured. All participants in the group will be followed up for 90 days to monitor the occurrence of falls and unplanned medical visits. Univariate and multivariate Logistic regression analyses will be adopted to explore the association between each index and the composite outcome, and a predictive model will be constructed. The discrimination of the model will be evaluated by the area under the receiver operating characteristic curve (AUC), and the calibration will be evaluated by the Hosmer-Lemeshow test. It is planned to develop an integer scoring system based on the final model coefficients to facilitate the rapid application by front-line nursing staff.

**Discussion:**

This study aims to integrate simple indicators reflecting muscle structure, strength, dynamic function and nutritional status to construct a short-term risk assessment tool suitable for daily use in nursing homes. If verified to be effective, this scoring tool can help caregivers identify high-risk individuals at an early stage, implement targeted interventions in a timely manner, thereby reducing the incidence of falls and related adverse events, and improving the quality of care in nursing homes and the safety of older adults.

## Background

The causes of falls are complex. Recent studies have shown that muscle weakness and malnutrition in older adults are important risk factors for falls (1, 2). The World Health Organization reports that people with weak muscles have a five times higher risk of falling than those with normal muscle strength (3). When muscle strength is insufficient, it can have adverse effects on daily walking activities and balance (4). A prospective cohort study in Japan involving 415 urban and rural residents aged 50 to 89 has shown that reduced grip strength can increase the risk of falls by 40% (5). Therefore, leg muscle groups and grip strength tests are often used to assess an individual’s physical condition (6). Calf circumference and grip strength are simple indicators for evaluating muscle mass and strength, and have been proven to be one of the powerful predictors of death and fall risk in older adults (7). However, the risk of falls does not only stem from static muscle mass and strength deficiency, but is also closely related to dynamic physical functional performance. The Up and Go (TUG) test, as a classic functional mobility assessment tool, can comprehensively reflect the lower limb muscle strength, balance ability and mobility stability of the subjects (8). Research shows that a test duration of more than 12 s is associated with increased fall risk in some studies (9); however, the widely accepted clinical cut off for impaired mobility is 14 s (10, 11), effectively compensating for the limitation of merely assessing the “stock” of muscles without being able to evaluate their “functional” applications. It is worth noting that maintaining muscle health depends on adequate nutritional support. In particular, protein-energy malnutrition can disrupt the balance between anabolism and catabolism, prompting the body to enter a negative nitrogen balance state characterized by muscle protein breakdown, which directly leads to rapid loss of muscle mass (12). Malnutrition can exacerbate muscle loss and frailty, and frailty individuals, due to functional limitations, are at risk of loss of appetite and weight loss, further exacerbating malnutrition (13). In this cycle, involuntary weight loss (such as a decrease of more than 5% within 3 months) serves as an objective and easily accessible indicator reflecting changes in nutritional status and is a sensitive sign of deteriorating nutritional status. It is not merely a simple change in the weight number. Rapid weight loss often indicates potential health problems (1) and has been widely proven to strongly predict adverse health outcomes such as falls and hospitalization (14). Therefore, we will integrate and evaluate the indicators that directly reflect muscle mass (calf circumference), muscle strength (grip strength), functional mobility (TUG), and dynamic nutritional status (weight change, appetite). Being able to capture the physiological essence of fall risk from multiple dimensions including the static and dynamic aspects of muscle structure, strength, function and nutrition in a more systematic and comprehensive way is expected to improve the accuracy and practicality of prediction. Based on this, this study proposes a rapid scoring system based on nursing accessibility indicators to make up for the deficiency of insufficient comprehensive consideration of physical function and nutritional dynamic changes in the existing fall risk assessment.

### Research purpose

This study aims to develop and validate a rapid care scoring tool based on accessible indicators such as calf circumference, grip strength, TUG test, weight change and appetite, for predicting the risk of falls or unplanned medical visits, among older adults in nursing homes within 90 days. Through prospective cohort observations, we aim to verify the efficacy of this score in short-term risk prediction and assess its feasibility in clinical nursing practice. The research hypothesis is that a multi-dimensional score combining muscle function and nutritional status can more accurately predict the high risk of falls or unplanned medical visits (such as emergency visits or unplanned hospitalizations) within 90 days in older adults than traditional methods.

## Method/design

### Research design

This study will adopt a prospective observational cohort study design. The investigation will be conducted in Binzhou City, Shandong Province from September 2026 to November 2026. A convenience sampling method will be adopted to select 10 practical cooperation bases of older adults care institutions affiliated with the research unit, covering nursing homes in the central urban area and towns. A survey was conducted on all older adults who met the inclusion criteria in the selected nursing homes. A cohort including older residents of older adults care institutions was established, and a baseline assessment was carried out and followed up for 90 days to ensure the representativeness of the sample. During this period, the relevant indicators of the research subjects were regularly measured and recorded by the nursing staff, and the occurrence of falls and follow-up medical treatment outcomes was monitored. Through longitudinal observation, the correlation and predictive value of each indicator with the target event are evaluated in a real nursing environment. Convenience sampling was chosen because it allows feasible recruitment within the available resources and time frame, and because the selected ten affiliated nursing homes cover a range of urban and suburban settings, providing a diverse sample of residents with varying care needs. Nevertheless, we acknowledge that convenience sampling may limit generalizability; this is addressed in the Limitations section. This protocol follows the SPIRIT (Standard Protocol Items: Recommendations for Interventional Trials) guidelines where applicable, and the completed SPIRIT checklist is provided as Supplementary material 1.

### Research subjects

*Inclusion population*: this study will recruit all eligible older residents from ten nursing homes. Mainly included in the standard package.

*Including*: age ≥65 years old; Have resided in an nursing homes for at least 1 month and agree to participate in the research; At the time of enrollment, they were able to understand and cooperate with basic physical measurements and questionnaire surveys. We will ensure that the sample is representative, including older adults of different genders, age groups, and with varying physical functions and cognitive states. The research plan conducts subgroup analyses based on age (65–79 years old and 80 years old and above), gender (male and female), cognitive function status (normal, mild cognitive impairment, cognitive impairment), etc., to explore the applicability of the scores in different subgroups of people.

*Exclusion criteria*: older adults with an expected lifespan of less than 3 months at the time of enrollment or those in the hospice care stage were excluded. Those who have been bedridden for a long time due to hand osteoarthritis or severe disability and are unable to stand or walk (as the risk assessment of falls for such individuals is of limited significance); Those with severe cognitive impairment who are unable to understand or cooperate with simple instructions; Incomplete clinical data and any other circumstances judged by the researcher as unsuitable for participation in this study (such as those who were recently admitted to hospital due to acute illness and have not yet recovered). For those excluded, we will record the reasons in the filtering log.

### Data collection

#### Baseline measurement

At the time of enrollment, each older adults will be evaluated by trained caregivers, including.

##### Sociodemographic and clinical characteristics

Personal information (age, gender, education level, etc.), previous history of falls, diagnosis of chronic diseases (confirmed based on medical records), and medication use.

##### Cognitive function assessment [mini-mental state examination (MMSE)]

The assessment will be conducted using the Chinese version of the Mini-Mental State Examination (15). The Cronbach’s alpha coefficient of the Chinese version of the MMSE scale was 0.833, indicating good internal consistency. The total score of this scale is 30 points. One point is awarded for each correct answer, and 0 points are given for incorrect or no answers. It covers orientation, memory, attention and calculation, recall ability and language function. Trained researchers conduct the work in a quiet environment. After the scoring is completed, the impact of educational attainment on the score will be fully considered. The critical values of MMSE are as follows: illiterate ≤19 points, primary education ≤22 points, and junior high school and above education ≤26 points (16).

##### Activities of daily living (ADL)

The Chinese version of the Barthel Index was used for assessment. This scale was developed by Mahoney and Barthel and is a standardized tool widely used to assess patients’ basic activities of daily living functions (17). The Chinese version of this scale has good structural validity and criterion validity, with Cronbach’s alpha coefficient ranging from 0.92 to 0.93. It has good applicability among older adults population in China (18). Ten basic activities of daily living (including eating, bathing, dressing, grooming, controlling bowel movements, controlling urination, using the toilet, moving around, walking and going up and down stairs, etc.) are evaluated. Each activity is scored from 1 to 15 points, with a total score ranging from 0 to 100 points. All items are scored based on the degree of assistance required to complete the activity. The higher the score, the stronger the independence. A score lower than 60 indicates that the individual has significant functional dependence in daily life and requires assistance from others (17, 18).

#### Main indicators

##### Calf circumference

All measurements were performed in the early morning after an overnight fast and voiding, with participants resting in the supine position for at least 5 min before measurement. Use a non-elastic soft mass ruler to measure the circumference (unit: centimeters) at the thickest part of the subject’s right calf circumference. The right calf was chosen for consistency, as previous studies have shown high correlation between right and left calf measurements in older adults without unilateral edema or amputation. In cases where the right calf is unavailable (e.g., amputation, severe wound), the left calf will be measured and recorded. Participants assume a supine position with their legs relaxed. Measure twice, accurate to 0.1 centimeters, and record the average value of the measurements on both sides. Calf circumference can reflect musculoskeletal mass and nutritional status. The lower the value, the more likely the muscle mass may be insufficient (19).

##### Grip strength

The peak grip strength of the subject’s dominant hand will be measured using a calibrated Jamar hydraulic grip dynamometer (unit: kilograms). Although the Asian Working Group for Sarcopenia recommends measuring both hands, to reduce participant burden and given that many residents have unilateral weakness (e.g., post-stroke),we measure only the dominant hand (or the stronger hand if the dominant hand is affected by stroke or injury). The maximumof three attempts is recorded. This practical approach is consistent with several large cohort studies in institutionalized older adults. The subject assumes a sitting or standing position, with the trunk upright, arms hanging down, elbow joints bent at a 90-degree Angle, and holds with maximum force for 3 s. Repeat the grip dynamometer on his dominant hand three times, with sufficient rest between tests. Record the maximum reading as the grip strength value. Grip strength is regarded as a simple representative indicator of overall muscle strength. A low value indicates insufficient strength: based on grip strength, it is stratified by gender and the quartile of body mass index (BMI). Male: BMI ≤ 24 kg/m^2^, grip strength ≤29 kg; BMI24.1-26 kg/m^2^, grip strength ≤30 kg; Bmi: 26.1-28 kg/m^2^, grip strength ≤30 kg; BMI > 28 kg/m^2^, grip strength ≤32 kg. Female: BMI ≤ 23 kg/m^2^, grip strength ≤17 kg; Bmi: 23.1–26 kg/m^2^, grip strength ≤17.3 kg; Bmi: 26.1–29 kg/m^2^, grip strength ≤18 kg; BMI > 29 kg/m^2^, grip strength ≤21 kg (20).

##### TUG test

The balance and movement functions of an individual are evaluated using the TUG test (10). This test is a widely validated reliable tool for assessing the dynamic balance, mobility and fall risk of older adults. The test was conducted in a quiet and open area. You need to prepare a standard-height chair (with a seat height of about 46 cm), a tape measure, a cone for marking and a stopwatch. Have the participants sit on the chairs, with their backs against the backrests and their hands on the armrests. A cone is placed 3 meters directly in front as a marker. At the beginning of the test, we issued standardized instructions to the subjects: when the tester shouted “Start,” the subjects were asked to stand up from the chair, walk to the other side of the marker at a speed that the subjects felt safe and normal, turn around and go around the marker, then walk back and sit down again. At the same time as the “Start” command was given, the researchers activated the stopwatch to keep time. The timing began the moment the participants’ buttocks started to leave the chair surface and ended when their buttocks touched the chair surface again. Record the time required to complete the entire task, accurate to 0.1 s. To ensure the reliability of the data, each participant took two tests with a one-minute break in between to avoid fatigue. In the subsequent analysis, the shortest time between the two tests will be taken as the final score of the participant. Based on the established clinical critical values, we defined a TUG test time of more than 14 s as impaired mobility function, indicating a relatively high risk of falls (10, 11).

##### Weight change

Weight change will be assessed over a standardized 3-month period prior to enrollment. For residents who have resided in the nursing home for more than 3 months, we will obtain the body weight measured approximately 90 days before enrollment (or the closest available measurement from medical records). For residents with a stay shorter than 3 months, the admission weight will be used as baseline, the actual time interval will be recorded and adjusted in sensitivity analyses. Clinically significant weight loss is defined as a decrease of >5% during that period (19, 21).

##### Appetite score (SNAQ)

It is an internationally recognized appetite assessment tool specifically developed for older adults. SNAQ is a rapid and simple self-assessment screening tool that can predict the risk of non-voluntary weight loss in older adults within half a year. It can be verified in the past without the need for trained assessors or laboratory tests (22). The simplified nutrition and appetite questionnaire was used to score the appetite of the subjects in the recent two weeks. The SNAQ consists of four questions, which, respectively, assess appetite, satiety, food taste and the number of meals per day. Each question is worth 5 points, and the total score ranges from 4 to 20 points. The lower the score, the worse the appetite. Studies have shown that ≤14 points can be used as the critical value for predicting malnutrition and involuntary weight loss (22, 23). The Chinese version of the SNAQ has been validated in community-dwelling older adults showing good reliability and validity for predicting involuntary weight loss (24, 44). However, validation specifically in institutionalized nursing home populations remains limited, and we acknowledge this limitation. Although the SNAQ is a validated screening tool, its reliance on self-report may introduce recall or social desirability bias. To mitigate this, we will administer the questionnaire in a standardized, quiet setting and cross-reference responses with nursing staff’s observations of food intake when available.

### Strategies to improve adherence

To enhance participant retention and adherence, regular follow-up reminders will be provided by nursing staff during routine care. Participants who complete the 90-day follow-up will receive a small health education booklet as a token of appreciation.

### Follow-up duration and endpoint events

A 90-day follow-up period was selected for several reasons. First, falls and unplanned medical visits within 90 days represent a clinically meaningful short-term risk window, allowing caregivers to implement timely preventive interventions. Second, a 90-day window aligns with several previous fall prediction studies, facilitating comparison of our findings. Third, shorter follow-up reduces loss to follow-up and recall bias, which is particularly important in nursing home settings where resident turnover is high. All the enrolled subjects will be followed up for at least 90 days. Fall events that occurred during the follow-up period (defined by the WHO as sudden, involuntary, and unintentional changes in body position, falling to the ground or a lower level, excluding falls caused by clear medical events such as acute stroke) (25) and unplanned medical visits events (defined as the need to visit a medical institution or be hospitalized due to any unplanned health problems) Including emergency visits or readmissions will be monitored as the primary outcome. The composite endpoint of falls or unplanned medical visits was chosen because both events represent significant clinical deterioration that often leads to increased care dependency and healthcare resource utilization. Additionally, the composite endpoint increases the number of events to support model development. However, we recognize that risk factors may differ. Therefore, secondary analyses will be performed with (1) falls alone and (2) unplanned medical visits alone as separate outcomes. The results of these secondary analyses will be interpreted cautiously. It should be noted that the risk stratification (high, medium, low) based on baseline scores is for descriptive and exploratory analyses only. The nursing staff providing daily care to residents will be blinded to the risk groups to prevent any differential care or monitoring that might influence outcome ascertainment. Risk classifications will not be shared with caregivers.

During the 90-day follow-up period, the above physical indicators were tracked and measured once a month to record the dynamic change trend. At the same time, caregivers in nursing homes should immediately report and register falls and follow-up medical visits: once an older adults falls, the date, location, injury condition and possible causes should be recorded immediately. During this period, if older adults person needs to go out for medical treatment or be hospitalized due to illness, the specific dates and reasons should also be recorded.

The research team regularly reviews the institution’s nursing records and medical records every 2 weeks to ensure that information on all target events is collected without omission. In addition to passive reporting, a standardized active monitoring protocol will be implemented: (1) weekly face-to-face or telephone check-ins with floor nurses to query any unreported falls or medical visits; (2) use of a structured fall/visit log form that includes date, time, location, circumstances, and injury severity; (3) monthly cross-validation of log entries against hospital admission records to capture out-of-facility events. Caregivers will be reminded regularly to report all events, regardless of perceived severity, to minimize underreporting. For those who have experienced multiple events, the number of events can be recorded, but the main analysis should take the first event as the outcome point.

Any adverse events occurring during the follow-up period, including falls resulting in injury or any other health deterioration requiring medical attention, will be recorded in the institution’s incident reports and reviewed by the research team weekly. All adverse events will be reported to the ethics committee in accordance with institutional guidelines.

**Figure 1:**
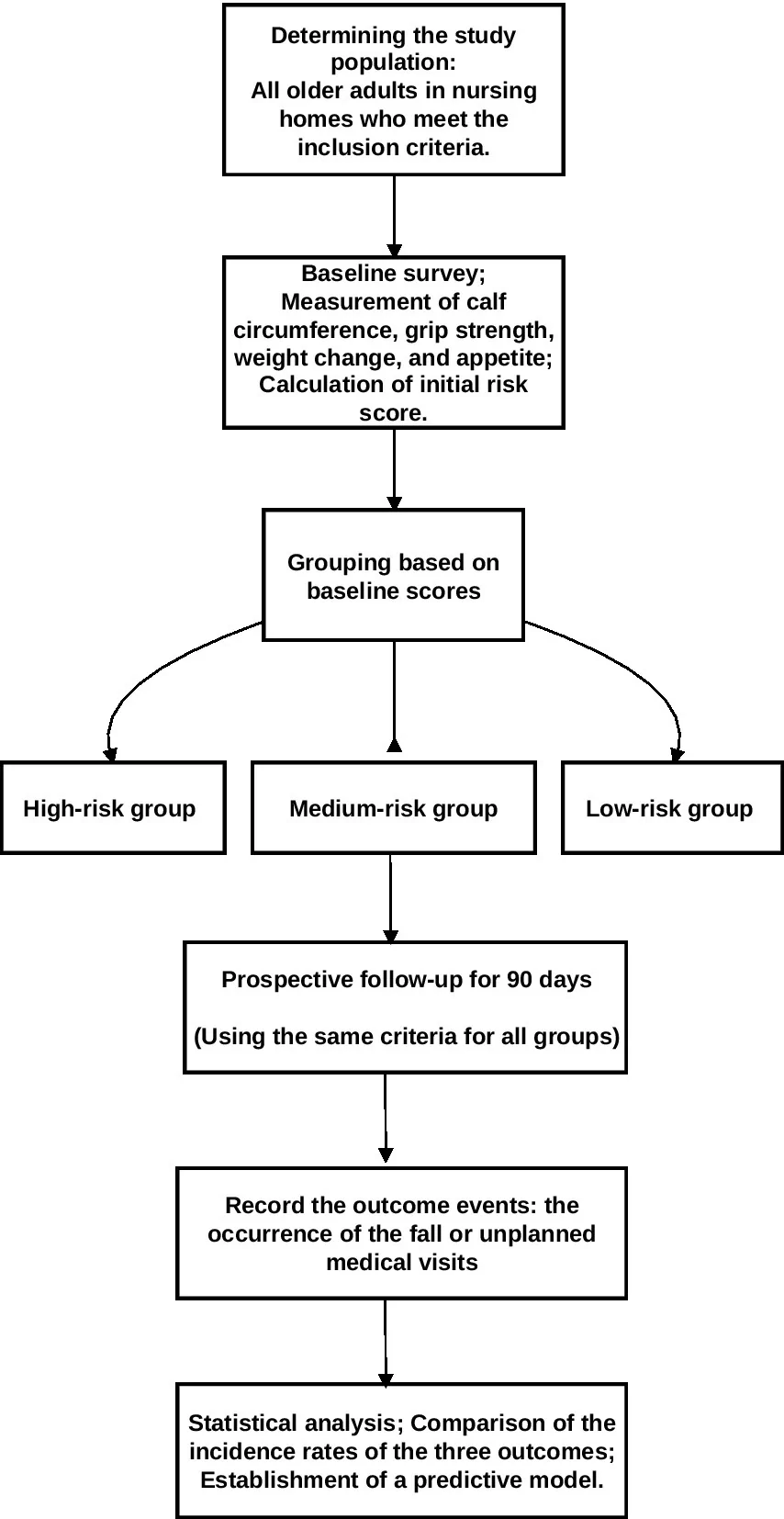
Study flow chart. The flowchart illustrates the process of participant recruitment, baseline assessment, 90-day follow-up, and outcome ascertainment. Caregivers are blinded to risk group assignments during the follow-up period.

Each subject was timed from the time of enrollment. The first fall or unplanned medical visits within 90 days was recorded as the occurrence of the outcome event. Those who did not have an outcome were considered as having no outcome at the end of the 90-day follow-up. For the occurrence time of the composite endpoint, our main concern is whether it occurred within the 90-day window period. The subsequent analysis takes this binary classification result as the dependent variable (see Figure 1).

### Variable settings

#### Predictive factors

This study focuses on the following five readily available indicators in nursing practice: ① Calf circumference: A continuous variable in centimeters, grouped according to the critical value < 31 cm in the literature. ② Grip strength: A continuous variable in kilograms, which will be classified as “low muscle strength” or “normal” based on the gender-specific threshold recommended by the Asian Sarcopenia Working Group. ③ TUG test: A continuous variable in seconds. Based on established clinical standards, the TUG test for more than 14 s indicates impaired mobility function and converts it into a binary categorical variable. ④ Percentage change in weight: The continuous variable calculated. A binary classification will be created to define “involuntary weight loss > 5% within 3 months prior to enrollment” as clinically significant weight loss. ⑤ Appetite: The simplified nutritional appetite questionnaire score (ranging from 4 to 20 points) was used as a continuous variable for assessment. The verified critical value (≤14 points) will be used to convert it into the binary variable of “anorexia.”

#### Outcome variables

The endpoint of whether a fall or unplanned medical visits occurred within 90 days. This is a binary variable (yes/No). If at least one fall event or one unplanned medical visit event (including emergency visits or readmission) occurs during the 90-day follow-up period, the outcome is recorded as “yes.”

#### Adjust covariates

To maintain a favorable events-per-variable ratio and avoid overfitting, the main multivariable model will include only three covariates (age, sex, and history of falls) in addition to the five primary predictors, giving a total of eight candidate variables. Age (65–79 years vs. ≥80 years), sex (male/female), and previous history of falls (yes/no) were selected because they are consistently identified as strong confounders in the fall prediction literature. Other potential confounders (e.g., cognitive function, ADL, number of chronic diseases) will be examined in univariable analyses but will not be forced into the multivariable model unless they show a strong association (*p* < 0.05) without causing multicollinearity. The selection of these covariates was based on the fall risk factors reported in the literature and our consideration of the characteristics of the study population (26–28). Including covariates helps control confounding bias, thereby more accurately assessing the independent predictive role of the primary independent variables.

### Quality control

This study can integrate index measurement into the routine nursing process without the need to add additional diagnosis and treatment procedures. The data recorded by the nursing staff was reused in the research, which not only improved the data utilization rate but also avoided repetitive work. To ensure measurement reliability and standardization, all participating nursing staff will complete a comprehensive training program before data collection. The training includes: (1) a two-day standardized session covering all measurement protocols (calf circumference, grip strength, TUG, weight, SNAQ), including the specification that calf circumference measurements are to be conducted in the early morning after fasting and voiding, with participants supine for 5 min; (2) instruction on proper use of calibrated equipment (Jamar dynamometer, electronic scale, stopwatch); (3) data recording and double-entry procedures. Competency will be assessed through practical demonstrations, and only staff who successfully demonstrate correct technique will be allowed to collect data. Equipment will be calibrated according to manufacturer specifications. Monthly supervisory visits will be conducted to monitor protocol adherence, and inter-rater reliability will be assessed by calculating the intraclass correlation coefficient (ICC) for a random 10% of measurements.

#### Sample size estimation

According to the sample size principle of Logistic regression modeling recommended in the literature, we need to ensure that each predictor variable has sufficient outcome events to stably estimate the model parameters. A common rule of thumb is that each independent variable should have at least about 10 events (EPV principle) (29). Assuming that the incidence of falls or unplanned medical visits within 90 days is approximately 20% (26, 30), the final multivariable logistic regression model will include 8 candidate variables (5 primary predictors + 3 covariates). This gives an events-per-variable ratio of 12.5, which exceeds the recommended minimum of 10. To account for an anticipated 15–20% loss to follow-up or missing data, we plan to recruit approximately 600 participants (31). If the actual number of enrolled participants is insufficient, it may be considered to appropriately streamline the model variables or extend the follow-up period to increase the number of events.

### Data management

To ensure high-quality data collection and management, we will use a professional electronic data capture platform meeting standard research requirements (e.g., RED Cap, Neo, or an internally developed secure database with equivalent functionality). The system will be implemented in collaboration with the university’s data center, which, as a pilot digital campus of the Ministry of Education, possesses the necessary infrastructure to support such a platform. Key quality control measures will include: (1) automatic validation rules (e.g., range checks: calf circumference 20–50 cm, grip strength 0–50 kg, TUG 0–120 s) and audit trails; (2) double data entry for a random 10% of the sample by a second researcher, with discrepancies resolved by referring to source documents; and (3) regular data cleaning and weekly backups. All data will be stored in a password-protected secure environment, and participant privacy will be protected by unique identification codes.

### Data analysis methods

#### Description of baseline characteristics

Statistical analysis in this study will be conducted using SPSS Statistics (Version 26.0) and R language (Version 4.3.0). Firstly, for baseline data, continuous variables that conform to the normal distribution will be expressed as mean ± standard deviation, those that do not conform to the normal distribution will be expressed as median (interquartile range), and categorical variables will be expressed as frequency (percentage). In the inter-group comparison, independent and sample t-tests or Mann–Whitney U tests or chi-square tests will be used to compare the baseline characteristics (corresponding to continuous variables and categorical variables, respectively) of participants with and without compound conjugations (falls/unplanned medical visits) to identify potential differences.

#### Development and verification of predictive models

The main analysis of this study is to construct a Logistic regression model for predicting the risk of falls /unplanned medical visits within 90 days. Each major indicator (calf circumference, grip strength, TUG test, percentage change in weight, SNAQ score) and all preset confounding factors (age, gender, MMSE score, history of falls, number of chronic diseases, ADL score) were, respectively, incorporated into the univariate Logistic regression model, and their unadjusted odds ratios and 95% confidence intervals were calculated. The variables with a *p* value < 0.1 in the univariate analysis were then jointly included in the multivariate Logistic regression model. We will adopt the backward step-by-step method (with the exclusion criterion being *p* > 0.05) to determine the final prediction model. The model results will report the adjusted odds ratio and its 95% confidence interval. Secondary analyses will use separate logistic regression models with falls and unplanned medical visits as independent outcomes.

#### Model performance evaluation

To address overfitting and provide optimism-corrected estimates, we will use bootstrap resampling (500 repetitions) for internal validation. The area under the receiver operating characteristic curve (AUC) and calibration slopes will be optimism-corrected. This study follows the TRIPOD (Transparent Reporting of a Multivariable Prediction Model for Individual Prognosis or Diagnosis) statement for prediction model development and internal validation. The Hosmer-Lemeshow test will be used to evaluate the consistency between the predicted probability of the model and the actual observed probability (*p* > 0.05 indicates good calibration).

#### Sensitivity analysis

Based on the coefficients of the multivariate Logistic regression model, we developed an integer scoring system to convert continuous variables into categorical variables and assign scores, thereby forming a rapid scoring tool that is convenient for use on the front line of care for older adults. Monthly measurements of calf circumference, grip strength, TUG, weight, and appetite were collected primarily for descriptive purposes to track changes over time. These repeated measures were not treated as time-varying covariates in the main logistic regression model, which used baseline values to predict the 90-day composite outcome. The monthly data will be used in a separate secondary analysis to explore longitudinal trends. Therefore, to test the stability of the model, we will, respectively, fit the models in the following preset subgroups and conduct interaction tests: age (65–79 years vs. ≥ 80 years), gender (male vs. Female), cognitive status (normal MMSE vs. impaired). If the missing rate of the main variable is greater than 5%, the multiple interpolation method of the chain equation will be adopted to create five complete datasets. The results of the model constructed based on the interpolated dataset were compared with those of the original complete case analysis as a sensitivity analysis.

### Ethics and data privacy

This study was submitted to and approved by the Academic Ethics Committee of Binzhou Polytechnic (approval number: 2025006). This research will be conducted strictly in accordance with the Declaration of Helsinki and relevant ethical standards in China to ensure compliance with ethical principles and the maximum protection of the rights and interests of the subjects. All subjects will receive comprehensive written and oral information regarding the purpose, process, potential risks and benefits, confidentiality policy, and the ability to withdraw from the study at any time without bearing any consequences before enrollment. All subjects are required to sign a written informed consent form. For the subjects who are unable to write, the research team will record in detail their oral consent.

Because this is an observational cohort study with no experimental intervention, a formal Data Safety Monitoring Board was not established. Nevertheless, any serious adverse events (e.g., falls resulting in fracture or hospitalization) will be reported to the institutional ethics committee within 24 h, and the study may be paused if unforeseen harm is detected.

## Discussion

This study aims to develop and validate a rapid scoring tool based on care accessibility indicators (calf circumference, grip strength, TUG, weight change and appetite) to predict the composite risk of falls or unplanned medical visits within 90 days among older adults in nursing homes. With the intensification of global population aging, falls and their related consequences have become a major public health issue affecting the health and quality of life of older adults, especially in the high-risk environment of nursing homes, the situation is even more severe (32). Although existing guidelines emphasize the importance of fall risk assessment, in the practical application of nursing homes there is still a lack of an assessment tool that can comprehensively reflect the multi-factorial nature of risks, is simple and easy to implement, and is suitable for routine use by non-professional caregivers (26, 33, 34). This study integrated core indicators reflecting muscle structure and function (calf circumference, grip strength), dynamic physical performance (TUG test), and changes in nutritional status (weight changes, appetite). This comprehensive assessment approach is highly consistent with the current understanding of the physiological mechanism of fall risk. Sarcopenia and frailty have been confirmed to be important factors in falls (35, 36). Calf circumference and grip strength, as simple indicators for evaluating muscle strength and muscle capacity, have had their predictive value confirmed in multiple studies (7, 37). The TUG test can dynamically measure lower limb muscle strength, balance and mobility stability. Its prolonged time consumption is significantly associated with an increased risk of falls (8, 38). More importantly, this study included two nutritional indicators: weight change and appetite. Involuntary weight loss and loss of appetite are often early signs of malnutrition (2, 13), and they further increase the occurrence of adverse events such as falls/unplanned medical visits by exacerbating muscle loss and weakness (39). Combining the nutritional dimension with the muscle function dimension can more systematically reveal the vicious cycle among “muscle - nutrition - function,” thereby providing more accurate risk prediction capabilities. The selected indicators are all simple measurements that nursing staff can obtain in their daily work, without relying on specialist evaluations or expensive equipment. We have developed a rapid scoring tool for nursing care, highlighting simplicity and real-time performance. It is convenient for front-line nursing care staff in nursing homes to implement and provide timely warnings for high-risk individuals. Nursing staff can calculate the risk level by looking up tables or scoring based on the measured values without a computer, truly achieving “quick scoring.” This is different from some models that require computer programs to calculate risk probabilities (40), and is more suitable for the grassroots environment, fully embodying the innovative practice led by nursing. Paying attention to the relatively short-term outcome prediction of 90 days can help identify high-risk individuals in a timely manner at the early stage when older adults move into nursing homes. Short-term risk scores can guide nursing staff to promptly take fall prevention measures or medical interventions and have strong practical value. Currently, most fall risk models focus on long-term risks of more than 1 year (41, 42, 43). At the same time, integrating falls and unplanned medical visits into a composite outcome makes the score not limited to fall prevention only. It also takes into account the risk of deteriorating health conditions requiring medical intervention (28), which more comprehensively reflects the safety and health risks of older adults in nursing homes and is in line with the concept of comprehensive care.

## Limitations

The use of convenience sampling from affiliated nursing homes may introduce selection bias, as facilities were not randomly selected. This limits the representativeness of the sample to the broader population of nursing homes, particularly those without academic affiliations. We have attempted to mitigate this by including both urban and suburban institutions and by clearly describing the characteristics of participating sites. Firstly, this study was conducted in ten nursing homes in Binzhou City, Shandong Province. Although we covered both urban and rural institutions, the samples still came from the same geographical area, which limits the generalizability of our findings to other regions and nursing homes with different cultural backgrounds. More importantly, these institutions were selected as “practical cooperation bases affiliated with the research unit,” introducing additional selection bias beyond the geographic limitation. Affiliated sites are likely to share similar care practices, staffing models, and patient populations due to their administrative or collaborative ties with the research unit. Such homogeneity may reduce the variability typically found across independently operated nursing homes and thus systematically affect external validity. Therefore, our results may not be directly applicable to non-affiliated or differently structured nursing home settings. Future research should include a broader range of nursing homes without such institutional affiliations to confirm the universality of our rapid scoring tool. Secondly, the outcome measures of this study were mainly collected through the care records of the nursing institutions and the self-reports of older adults or their caregivers. Despite the measures we have taken, such as regular case checks, this approach still cannot completely avoid recall bias or reporting omissions, especially for fall incidents that do not cause obvious injuries. In future research, the automatic capture of electronic medical records will be utilized to obtain a better data collection method. Thirdly, for residents with a short length of stay, the standardization of the weight change assessment period may be imperfect; we will record the actual time interval and address this in sensitivity analyses. Finally, although we limited the number of covariates to avoid overfitting, unmeasured confounders (e.g., medication use, environmental hazards) may still exist. The presence of these unmeasured confounding factors may affect the absolute accuracy of the prediction. Therefore, the aim of this study is to develop a rapid and early screening tool rather than a precise comprehensive diagnostic instrument.
