# Supplementary material for: Predicting 90-day falls and unplanned medical visits in nursing home residents using rapid nursing scores including calf circumference, grip strength, Timed Up and go (TUG) test, weight change, and appetite: a prospective observational cohort study protocol
Source: Front Public Health. 2026 Aug 6;14:1812292. doi: 10.3389/fpubh.2026.1812292 (PMC13491651; doi:10.3389/fpubh.2026.1812292)
Supplement: Supplementary file 1 [file Supplementary_file_1.pdf]

Chapter/Entry	nu mb er	Entry Description	Report Location (Page Number/Line Number)	Description (if not applicable or not reported)
title	1	Title describing the study design, subjects, and intervention measures (including trial abbreviations if applicable).	Rows 1 – 3	
Trial Registration	2	Name and registration number of the trial registration authority. If not registered, specify the name of the institution planning to register	Not reported	Upon editorial confirmation, this study is an observational study and does not require clinical trial registration.
Solution Version	3	Version number and date of the plan	Not reported	This study does not require submission
aid financially	4	Sources and types of funding, supplies, and other support	Lines 609 – 611	
Roles and	5a	Name, Organization, and Role of	Rows 5 – 15;	

Chapter/Entry	number	Entry Description	Report Location (Page Number/Line Number)	Description (if not applicable or not reported)
Responsibilities		the Solution Contributor	Lines 623 – 631	
	5b	Name and contact information of the trial initiator	Line 25; Line 95;	Initiator: Binzhou Vocational College
	5c	The roles of the study investigators and funders (if applicable) in research design, data collection, management, analysis, interpretation, report writing, and submission decision-making.	Lines 610 – 611	The funding organization did not participate in any aspect of the research.
	5d	The composition, roles, and responsibilities of the Coordination Center, Steering Committee, End-Point Determination Committee, Data Management Team, and other trial monitoring institutions or individuals	**Not reported**	No independent oversight committee was established; this is an observational low-risk study. The decision has been made not to supplement the data.

Chapter/Entry	number	Entry Description	Report Location (Page Number/Line Number)	Description (if not applicable or not reported)
**Introduction**				
Background and Theoretical Basis	6a	Description of the research question and rationale for conducting the experiment, including a summary of relevant studies.	Lines 47 – 82	
	6b	Explanation of the selected comparison	not applicable	**N/A: Observational cohort study, without control group**
purpose	7	Specific research objectives or hypotheses	Lines 83 – 91	
**Method: Design**				
experiment design	8	Description of the trial design, including trial type, allocation ratio, and framework	Lines 92 – 111	Prospective observational cohort study

Chapter/Entry	number	Entry Description	Report Location (Page Number/Line Number)	Description (if not applicable or not reported)
**Methods: Study subjects, intervention measures, and outcome indicators**				
Research Site	9	Description of the research sites and list of countries where data were collected	Lines 95 – 98	10 elderly care institutions in Chinas Shandong Province
Selection Criteria	10	Inclusion and exclusion criteria for study subjects	Lines 112 – 130	
intervention study	11a	Detailed information on the intervention measures for each group is sufficient to conduct replication studies.	not applicable	N/A: Observational study, no intervention
	11b	Standards for terminating or modifying assigned interventions	not applicable	N/A: No intervention measures
	11c	Strategies to improve adherence to intervention programs	not applicable	N/A: No intervention measures

Chapter/Entry	number	Entry Description	Report Location (Page Number/Line Number)	Description (if not applicable or not reported)
	11d	Relevant accompanying care and intervention measures permitted or prohibited during the trial period	not applicable	N/A: No intervention measures
Outcomes Indicator	12	Primary, secondary, and other outcome indicators, including specific measurement variables, assessment metrics, aggregation methods, and time points.	Lines 131 – 220	Primary outcome: A composite outcome of falls and unplanned medical visits within 90 days
Research Subject Timeline	13	Timetable for inclusion, intervention, evaluation, and visits	Lines 273 – 277	Technical roadmap provided
Sample Size	14	Estimate the required number of participants and determine the methodology needed to achieve the research objectives.	Lines 325 – 328	EPV principle: estimated sample size N = 500 – 600

Chapter/Entry	number	Entry Description	Report Location (Page Number/Line Number)	Description (if not applicable or not reported)
recruit	15	Recruitment strategy to achieve the target sample size	Lines 94 – 98	From a convenient sample of 10 institutions...
**Method: Intervention Allocation**				
distribution	16a	Method for generating an allocation sequence	not applicable	N/A: Observational study, without randomization
	16b	Mechanism for implementing the allocation sequence	not applicable	N/A: Observational study, without randomization
	16c	Who generates the allocation sequence, who recruits study subjects, and who assigns them to each group?	not applicable	N/A: Observational study, without randomization
blind method	17a	Who gets blinded after allocation?	not applicable	N/A: No intervention measures implemented; no blinding involved

Chapter/Entry	number	Entry Description	Report Location (Page Number/Line Number)	Description (if not applicable or not reported)
	17b	If a blind method is implemented, under what circumstances is blinding removal permitted?	not applicable	N/A: Blinding was not implemented
**Methods: Data collection, management, and analysis**				
Data Collection Methods	18a	Plan for the evaluation and collection of outcome indicators, baseline data, and other trial-related data	Lines 131 – 220	The measurement methods for each indicator are described in detail.
	18b	Improve the retention rate of study subjects and implement the follow-up completion plan.	Lines 222 – 225	Supplemented with: routine follow-up reminders, health education handbook
data management	19	Data Entry, Coding, Privacy Protection, Storage, and Backup Plan	Lines 337 – 347	

Chapter/Entry	number	Entry Description	Report Location (Page Number/Line Number)	Description (if not applicable or not reported)
statistical method	20a	Statistical methods for analyzing primary and secondary outcome indicators	Lines 348 – 369	
	20b	Additional Analytical Methods	Lines 370 – 387	
	20c	Analysis population and methods for handling missing data	Lines 384 – 387	Multiple interpolation is employed when the missing rate exceeds 5%.
Method: Monitoring				
Data Monitoring	21a	Composition, Responsibilities, and Reporting Structure of the Data Monitoring Committee	not applicable	N/A: Observational low-risk study without a data monitoring committee established
	21b	Description of the interim analysis and early termination guidelines	not applicable	N/A: No interim analysis is planned.
adverse	22	Plan for the collection, evaluation,	Lines 397 – 400	Supplemented: Weekly review, event

Chapter/Entry	number	Entry Description	Report Location (Page Number/Line Number)	Description (if not applicable or not reported)
event		reporting, and management of adverse events		reporting, and submission to the ethics committee.
audit	23	Frequency and procedures of audits conducted during the implementation of the trial	not applicable	**N/A: No independent audit is planned**
**Ethics and Communication**				
Research Ethics Approval	24	Plan seeking approval from the Research Ethics Committee/Institutional Review Board	Lines 389 – 390;<br>Lines 594 – 599	Approved by the Academic Ethics Committee of Binzhou Vocational College (Approval No.: 2025006)
Plan Revision	25	Notice Plan for Significant Changes to Key Policies	not applicable	This study is an observational cohort study and a non-interventional trial, with an existing clinical trial registration number (ChiCTR2500110619).

Chapter/Entry	nu mb er	Entry Description	Report Location (Page Number/Line Number)	Description (if not applicable or not reported)
informed consent	26a	Who will obtain the informed consent or authorization from the potential study subject or their authorized representative?	Lines 115 – 122	
	26b	Additional consent provisions for the collection and use of study subject data and biological specimens	not applicable	**N/A: Not applicable to biological specimen collection**
confidentialit y	27	How to collect, share, and maintain personal information of potential and enrolled study participants	Lines 309 – 336	Password Protection, Unique Identification Code
conflict of interest	28	The principal investigators financial and other competitive interests	Lines 603 – 604	Authors Statement: No Conflict of Interest
Data	29	Statement regarding who will have	Lines 614 – 618	Added: Reasonable requests, ethical

Chapter/Entry	number	Entry Description	Report Location (Page Number/Line Number)	Description (if not applicable or not reported)
Acquisition		access to the final trial dataset		approval
Assistance and Post-Treatment Care	30	If applicable, relevant provisions regarding support services and post-test care	not applicable	N/A: Observational study; does not involve post-treatment care. Scales and measurement tools are described in the main text.
Communication Policy	31a	The plan of researchers and initiators to disseminate the trial results	Not reported	The manuscript does not separately describe the plan for disseminating the research findings (not supplemented during the study protocol phase).
	31b	Author Signature Eligibility Guide	Lines 620 – 628	The CRediT author contribution statement has been provided.
	31c	If applicable, plan to provide the	Lines 614 – 618	Decidentified data can be requested

Chapter/Entry	number	Entry Description	Report Location (Page Number/Line Number)	Description (if not applicable or not reported)
		public with a complete proposal, participant-level datasets, and statistical code.		appropriately
appendix				
Informed Consent Materials	32	Template for the informed consent form and other relevant documents provided to participants	supplemental instrument	The informed consent form template will be uploaded as a supplementary document
biological specimen	33	Collection, laboratory evaluation, and storage planning for biological specimens	not applicable	N/A: Not applicable to biological specimens
intellectual property right	34	ownership of intellectual property rights and any restrictions on publication	not applicable	N/A: Academic research initiated by the investigator, without commercial funding or intellectual property disputes.
